# A novel *ATP1A2* mutation in a patient with hypokalaemic periodic paralysis and CNS symptoms

**DOI:** 10.1093/brain/awy283

**Published:** 2018-11-12

**Authors:** Marisol Sampedro Castañeda, Edmar Zanoteli, Renata S Scalco, Vinicius Scaramuzzi, Vitor Marques Caldas, Umbertina Conti Reed, Andre Macedo Serafim da Silva, Benjamin O’Callaghan, Rahul Phadke, Enrico Bugiardini, Richa Sud, Samuel McCall, Michael G Hanna, Hanne Poulsen, Roope Männikkö, Emma Matthews

**Affiliations:** 1 MRC Centre for Neuromuscular Diseases, Department of Molecular Neuroscience, UCL Institute of Neurology, Queen Square, London, UK; 2 Departamento de Neurologia, Faculdade de Medicina da Universidade de São Paulo (FMUSP), São Paulo, Brazil; 3 Division of Neuropathology, UCL Institute of Neurology, London, UK; 4 Neurogenetics Unit, UCL Institute of Neurology, Queen Square, London, UK; 5 DANDRITE, Nordic EMBL Partnership for Molecular Medicine, Aarhus University, DK-8000 Aarhus, Denmark

**Keywords:** hypokalaemic periodic paralysis, Na^+^/K^+^-ATPase, Na^+^/K^+^-pump

## Abstract

Hypokalaemic periodic paralysis is a rare genetic neuromuscular disease characterized by episodes of skeletal muscle paralysis associated with low serum potassium. Muscle fibre inexcitability during attacks of paralysis is due to an aberrant depolarizing leak current through mutant voltage sensing domains of either the sarcolemmal voltage-gated calcium or sodium channel. We report a child with hypokalaemic periodic paralysis and CNS involvement, including seizures, but without mutations in the known periodic paralysis genes. We identified a novel heterozygous *de novo* missense mutation in the *ATP1A2* gene encoding the α2 subunit of the Na^+^/K^+^-ATPase that is abundantly expressed in skeletal muscle and in brain astrocytes. Pump activity is crucial for Na^+^ and K^+^ homeostasis following sustained muscle or neuronal activity and its dysfunction is linked to the CNS disorders hemiplegic migraine and alternating hemiplegia of childhood, but muscle dysfunction has not been reported. Electrophysiological measurements of mutant pump activity in *Xenopus* oocytes revealed lower turnover rates in physiological extracellular K^+^ and an anomalous inward leak current in hypokalaemic conditions, predicted to lead to muscle depolarization. Our data provide important evidence supporting a leak current as the major pathomechanism underlying hypokalaemic periodic paralysis and indicate *ATP1A2* as a new hypokalaemic periodic paralysis gene.

## Teaser

Sampedro Castaneda *et al.* describe a boy with hypokalaemic periodic paralysis (hypoPP) and epilepsy without mutations in known risk genes. They show that a *de novo* mutation in *ATP1A2*, encoding a Na^+^/K^+^-ATPase subunit, gives rise to an inward cation leak current analogous to leak currents seen in other hypoPP cases.

## Introduction

Hypokalaemic periodic paralysis (hypoPP) is characterized by episodes of flaccid skeletal muscle paralysis accompanied by low serum potassium that typically occur in the early morning and last a minimum of hours. The characteristic age of onset is in the early teens. Paralysis occurs because the muscle membrane undergoes sustained depolarization that renders it inexcitable (Jurkat-Rott *et al.*, 2009).

Mutations in *CACNA1S* and *SCN4A* account for 80% and 10% of hypoPP cases, respectively, while 10% of cases remain genetically undefined (Matthews *et al.*, 2009). These genes code for the alpha subunits of voltage-gated calcium and sodium channels Ca_v_1.1 and Na_v_1.4, essential for excitation-contraction coupling and sarcolemmal excitability. HypoPP mutations affect arginine residues in the voltage sensing domains (VSDs) of both channels (Matthews *et al.*, 2009). These mutations have minor effects on normal channel activity but introduce a leak current through the VSDs, known as a ‘gating pore’ current. This anomalous cation current (Sokolov *et al.*, 2007; Struyk and Cannon, 2007) is typically active at resting membrane potential and conducts a net inward current that depolarizes the muscle, particularly in hypokalaemic conditions where the hyperpolarizing potassium current through the inward rectifier potassium channel K_ir_2.1 is reduced (Suetterlin *et al.*, 2014). Direct loss-of-function of K_ir_2.1 channels due to mutations in the *KCNJ2* gene can also lead to periodic paralysis with hypokalaemia in Andersen-Tawil syndrome (Plaster *et al.*, 2001).

The Na^+^/K^+^-ATPases convert ATP energy into steep electrochemical gradients for Na^+^ and K^+^ ions across the plasma membrane (Morth *et al.*, 2007; Clausen *et al.*, 2017). These gradients are essential for the maintenance of the resting membrane potential, the generation of electrical impulses and for driving secondary transport. The α2 subunit is expressed in glial cells of the CNS and in cardiac and skeletal muscle (Orlowski and Lingrel, 1988; Radzyukevich *et al.*, 2013; Illarionova *et al.*, 2014). To date, mutations in *ATP1A2* have been associated with familial and sporadic hemiplegic migraine (FHM and SHM) and alternating hemiplegia of childhood (AHC) (Bassi *et al.*, 2004; Bøttger *et al.*, 2012; Pelzer *et al.*, 2017), but skeletal muscle presentations have not been reported.

Here, we describe the clinical features and functional consequences of a novel *ATP1A2* mutation found in a young male with hypoPP lacking mutations in the known associated genes (*CACNA1S*, *SCN4A* or *KCNJ2*).

## Materials and methods

### Genetic analysis

Sanger sequencing of *CACNA1S* and *SCN4A* (for common hypoPP mutations) and the coding region of *KCNJ2* was carried out as previously described (Matthews *et al.*, 2009). Focused exome sequencing was performed using the Agilent Sure Select Focused Exome according to the manufacturer’s protocol. Sanger sequencing was used to analyse the presence of the *ATP1A2* variant in the proband and their parents. All clinical procedures were undertaken as part of routine clinical care.

### Molecular biology

Plasmids encoding the α2 and β1 subunits of the human Na^+^/K^+^-ATPase were used. The ‘wild-type’ α2 plasmid contained the mutations p.Q116R and p.N127D to reduce ouabain sensitivity (Price and Lingrel, 1988). The p.S779N mutation was introduced by site-directed mutagenesis (QuickChange, Agilent Technologies) and confirmed by sequencing the whole insert. *ATP1A2* (α2) and *ATP1B* (β1) mRNAs were transcribed using the mMessage mMachine kit (Ambion).

### Oocyte preparation

*Xenopus laevis* oocytes were obtained following procedures approved by the UK Animals (Scientific Procedures) Act 1986. Cells were defolliculated with Collagenase A (Roche) 2 mg/ml in oocyte Ringer and stored in modified Barth’s Solution supplemented with penicillin (50 U/ml), streptomycin (50 µg/ml) and amikacin (100 µg/ml) at 14–18°C. Oocytes were injected with a mix of *ATP1B* (2 ng) and wild-type or mutant *ATP1A2* (10 ng) mRNAs.

### Two-electrode voltage clamp

Two-electrode voltage clamp is routinely used to characterize functional properties of Na^+^/K^+^-ATPase pumps (Horisberger and Kharoubi-Hess, 2002; Li *et al.*, 2006; Poulsen *et al.*, 2010; Vedovato and Gadsby, 2014; Hilbers *et al.*, 2016). Data were collected with a GeneClamp 500B amplifier, Digidata 1200 digitizer and pCLAMP^™^ software (Molecular Devices) at room temperature. Currents were measured 2–4 days after injection in oocytes preincubated (>30 min) with a Na^+^-loading buffer (Poulsen *et al.*, 2010), unless otherwise mentioned. Electrode resistance was 0.2–0.7 MΩ when filled with NaCl 3 M to allow fast voltage clamp. Currents were elicited with 200-ms test steps from −160 to +60 mV, at 20-mV increments, from a holding potential of −30 mV. Recordings were sampled at 5 kHz and filtered at 1 kHz. Ouabain 1 µM was included in all recording solutions to block endogenous pumps.

Transient sodium-dependent currents were recorded in 0 [K^+^]_o_ (in mM: NaOH 115, sulphamic acid 110, MgCl_2_ 1, CaCl_2_ 0.5, BaCl_2_ 5, HEPES 10, pH 7.4) with and without 10 mM ouabain, and isolated offline by subtraction. Scale was maximized to minimize signal clipping, and 10 traces were averaged to improve signal-to-noise ratio. The integral of transient ouabain-sensitive currents at −30 mV following the test voltage steps was plotted against test voltage and fitted to a Boltzmann function:

(1) $$f(V)=\frac{A1-A2}{\left(1+e^{\frac{V-V½}{dV}}\;\right)}+A2$$

yielding the mid-point potential (V_1/2_), slope factor (dV) and top (A1) and bottom (A2) asymptotes. Total charge transfer, Q_tot_, represents the span of the Boltzmann fit (A1 − A2). Rate constants of Na^+^ binding/unbinding reactions derived from single (wild-type) or double (p.S779N) exponential fits to transient currents at the onset of the pulse. For p.S779N, the fast time constant, describing >90% of charge transfer, was analysed. Boltzmann fits to mean relaxation-voltage curves yielded the maximal forward and backward rate constants.

Leak currents were measured as the mean ouabain-sensitive currents in the last 50 ms of the test pulse_._ In some experiments, pH was altered or Na^+^ was replaced by NMDG^+^ as monovalent cation in a 3:1 ratio. In experiments with 145 mM Na^+^, 30 mM NaOH was added to 0 [K^+^]_o_ solution. Reversal potential and slope conductance were obtained from linear fits to the current-voltage data.

**F**orward pumping activity was measured in various [K^+^]_o_ concentrations before and after addition of 10 mM ouabain. Records were subtracted offline. In these experiments, extracellular NaOH was replaced with equimolar KOH. Concentration-response curves were fit with a Hill equation:

(2) $$\text{I }\left(\text{steady state}\right)=\text{A}1+\frac{\text{A}2-\text{A}1}{1+{\left(\frac{\left[\text{K}+\right]\text{o}}{\text{EC}50}\right)}^{\text{h}}}$$

yielding [K^+^] EC_50_ values, Hill coefficient (h) and top (A1) and bottom (A2) asymptotes.

### Statistical analysis

Analysis was performed using Clampfit 10.7, OriginPro 2016 and GraphPad Prism 4. Fit-derived parameters were compared with Student’s *t*-tests or one-way ANOVA. Current-voltage curves were compared with a two-way ANOVA, where the dependent variable was ‘current’ and the independent variables ‘voltage’ and ‘gene variant’. Data are presented as mean ± standard error of the mean (SEM) with significance level at *P < *0.05.

### Data availability

The authors confirm that the data supporting the findings of this study are available within the article and its Supplementary material.

## Results

### Clinical presentation

**Figure 1:**
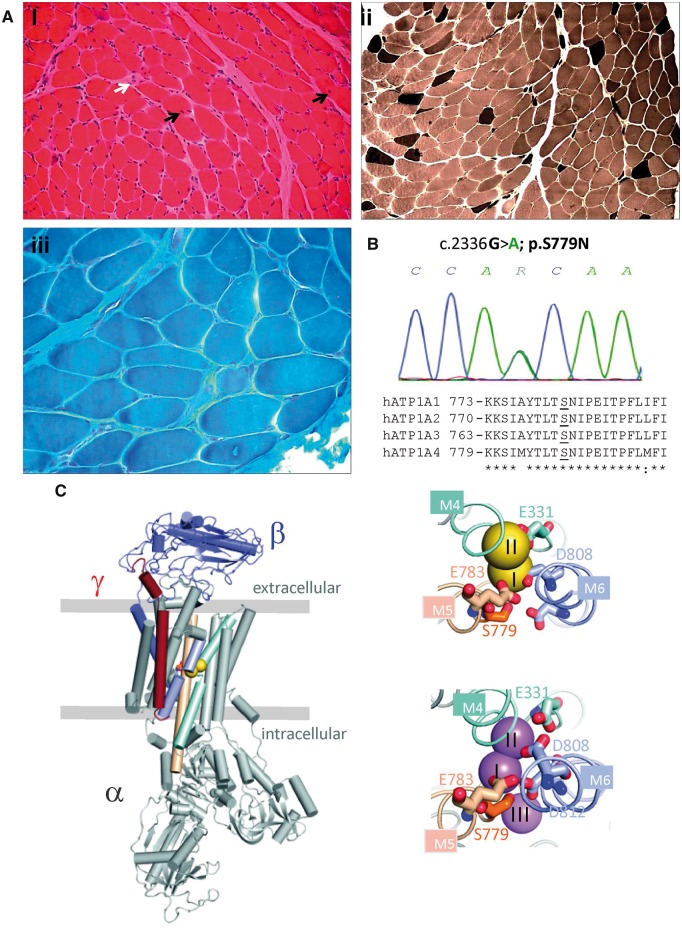
**Muscle pathology and genetic analysis of the hypoPP patient.** (**A**) Histological examination of a biceps brachii muscle biopsy performed at age 2 years and 3 months. (**i**) Haematoxylin and eosin staining showed variation in fibre diameter, increased internal nuclei (black arrows), a necrotic fibre (white arrow) and subtle endomysial fibrosis (also seen in **iii**) (**ii**) Myosin ATPase histochemistry at pH 9.4 indicated type I fibre predominance (pale stained fibres). (**iii**) Gomori trichrome staining. (**B**) Sequence chromatogram demonstrating the heterozygous mutation c.2336 G>A; p.S779N in the proband (above) and sequence alignment of human Na^+^/K^+^-ATPase alpha subunits. *ATP1A2*-S779 and analogous serine residues are underlined. (**C**) Structural context of S779. *Left*: An overview of the Na^+^/K^+^-ATPase structure with the alpha subunit in grey, the beta subunit in blue and the gamma subunit in red. The two potassium ions are yellow spheres, S779 is in orange stick, and the ion-coordinating transmembrane helices are light cyan (M4), wheat (M5) and light blue (M6). The boundaries of the membrane are indicated by horizontal lines. *Right*: Close-ups of the ion binding sites viewed from the extracellular side, top with two potassium ions (yellow), bottom with three sodium ions (violet). S779 is close to ion binding sites I and III. The figure was made using PDB structures 2ZXE (potassium bound) and 3WGU (sodium bound).

A 9-year-old Brazilian boy presented with episodes of flaccid muscle paralysis. Pregnancy and birth history were unremarkable. He experienced absence seizures with ocular aversion from age 4 months. These were initially resistant to treatment with status epilepticus occurring at age 1 year but eventually controlled with a combination of sodium valproate and levetiracetam. Seizures recurred with increasing age and increased drug doses were required. He had delayed motor milestones, not walking independently until 2 years 9 months, and speech delay with learning difficulties. At age 2 years he woke with tetraparesis and dysphagia lasting several hours, concomitant with a lower respiratory tract infection. Symptoms resolved with the infection and no specific diagnosis was made. He had two similar episodes at ages 3 and 4 years lasting several days. Episodes became increasingly frequent, averaging 5–10 per month. Paralysis attacks were bilateral, not associated with migraine or headache, occurred usually on waking and lasted hours to days. Triggers included carbohydrate meals and viral illness. Creatine kinase ranged from 382 to 1793 IU/l. Serum potassium was low (2.4 mM) during symptoms. Routine electrocardiogram was normal. A muscle biopsy undertaken shortly after an attack showed evidence of a non-specific myopathic process (Fig. 1A). Brain MRI was normal at 5 months but at 7 years demonstrated increased prominence of the ventricular system with bilateral mesial temporal sclerosis. EEG initially demonstrated epileptic activity although normalized on treatment. Routine EMG and nerve conduction study were normal but compound muscle action potential during an attack was significantly reduced compared to post-attack level, confirming peripheral impairment of neuromuscular function. Symptoms of paralysis improved with potassium supplements but were significantly worsened by acetazolamide.

There was no family history of similar symptoms, epilepsy or migraine.

#### Genetic analysis

No mutations were found in the *CACNA1S*, *SCN4A* or *KCNJ2* genes. A novel missense variant, c.G2336A, was identified in the *ATP1A2* gene, affecting a highly conserved residue (p.S779) of the α2 subunit of Na^+^/K^+^-ATPase (Fig. 1B and C) in an ion binding site where Na^+^ or K^+^ interact with Na^+^/K^+^-ATPases, depending on the conformation (Fig. 1B and C). This variant is absent in over 120 000 exomes from the Genome Aggregation Database (gnomAD). The asymptomatic parents did not carry the variant, suggesting it had arisen *de novo.*

### Functional characterization of p.S779N Na^+^/K^+^-ATPase

#### Anomalous leak current and sodium affinity

**Figure 2:**
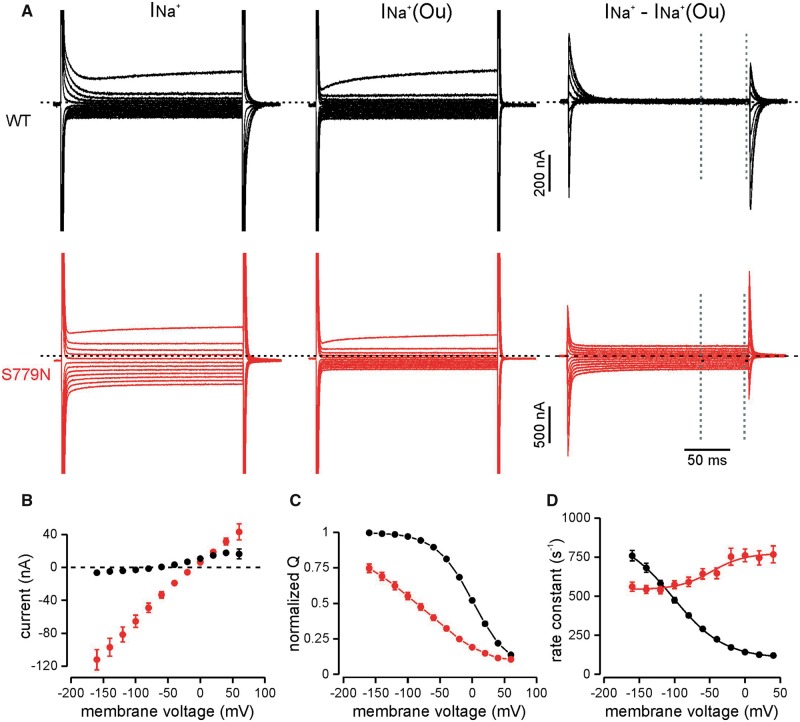
**Steady state and transient Na^+^ currents of WT and p.S779N pumps in the absence of extracellular K^+^.** (**A**) Representative raw current traces in absence (*left*) and presence (*middle*) of 10 mM ouabain and the ouabain-sensitive currents (*right*) in wild-type (black) and p.S779N (red) pumps in response to voltage steps from −160 to +60 mV, in 20-mV increments. Steady state currents at each voltage were measured in the last 50 ms of the 200 ms stimulus, indicated by dotted lines. (**B**) Average ouabain-sensitive steady state leak currents. Slope conductance between −140 mV and 0 mV was 0.75 ± 0.08 nA/mV for the p.S779N pump (*n = *27) while it was close to 0 for the wild-type pump (*n = *29). (**C**) Charge-voltage relationships. Na^+^ charge transfer was determined from the integral of the first 50 ms of the current trace at −30 mV following the steps to test voltages. Individual QV curves were fit by a standard Boltzmann function and normalized to their respective fits. V_1/2_: p.S779N −95.4 ± 7.1 mV, *n = *25; wild-type 2.6 ± 1.7 mV, *n = *29; slope: p.S779N 66.8 ± 4.8 mV, wild-type 28.9 ± 0.7 mV, *P < *10^−5^, unpaired *t*-test. (**D**) Rate constants of transient currents at the onset of the stimulus. Solid line represents a Boltzmann fit and yielded the overall forward/backward rate constants (p.S779N, 773.9 ± 27.7/542.6 ± 12.7 s^−1^, *n = *22 wild-type 102.3 ± 3.6/882.2 ± 32.9 s^−1^, *n = *26; *P < *0.0001, unpaired *t*-test).

The extracellular Na^+^ affinity was studied by analysing the release and rebinding of Na^+^ ions to the pump in absence of extracellular K^+^. Transient ouabain-sensitive Na^+^ currents in response to a series of voltage steps rapidly declined to zero for the wild-type pump. In contrast, the p.S779N ATPase carried an abnormal ouabain-sensitive steady state current following the Na^+^ transients (Fig. 2A and B). This leak component was voltage-independent and reversed at −12.7 ± 1.0 mV (*n = *27). Its amplitude was −49.2 ± 5.4 nA at −80 mV compared to a wild-type leak of −1.5 ± 0.9 nA (*n = *28).

The voltage dependence of transient currents was left-shifted in mutant pumps by almost 100 mV (Fig. 2C; *P < *10^−5^) and was less steep than in wild-type pumps (*P < *10^−5^), indicating that stronger hyperpolarizations are needed to reload Na^+^ binding sites and reflecting a reduced extracellular Na^+^ affinity of p.S779N. Additionally, the rate constants of relaxation of transient Na^+^ currents showed little voltage-dependence for p.S779N, while for the wild-type pump they decreased significantly with depolarization (Fig. 2A and D). Compared to the wild-type Na^+^/K^+^-ATPase, both forward and backward reaction rates, at depolarized and hyperpolarized voltages respectively, were significantly altered in the mutant (*P < *0.0001).

#### Reduced turnover rate and apparent K^+^ affinity

**Figure 3:**
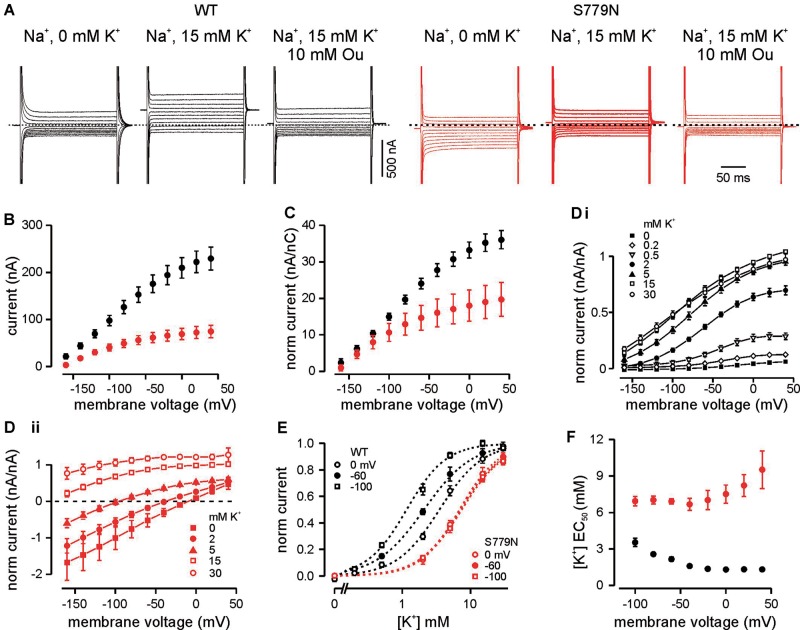
**Forward pumping currents of WT and p.S779N α 2 at various extracellular [K^+^]_o_.** (**A**) Example current traces of wild-type (black) and mutant (red) pumps in response to 200 ms steps from −160 to +40 mV, from a holding voltage of −30 mV in absence (*left*) and presence (*middle*) of 15 mM K^+^, and in presence of 15 mM K^+^ and 10 mM ouabain (*right*). K^+^-induced pump currents were isolated off-line by subtraction of currents in the presence of ouabain 10 mM. (**B**) Current-voltage relationship of steady state ouabain-sensitive currents measured in last 50 ms of each step (p.S779N *n = *17; wild-type *n = *20; *P < *0.0001 two-way ANOVA). (**C**) Current-voltage relationship of ouabain-sensitive currents normalized to total Na^+^ charge transfer (Q_tot_) in absence of K^+^, as a measure of functional protein expression (p.S779N *n = *7; wild-type *n = *13; *P = *0.001, two-way ANOVA). Q_tot_ was estimated in a subset of cells where lower amplifier gain was used to minimize current saturation because of capacitive artefacts. Ten traces were averaged to reduce the noise and then used to determine the span of the Boltzman curve (Q_tot_: S779N 5.4 ± 0.9 nC, *n = *7; wild-type 5.5 ± 0.7 nC, *n = *13). (**D**) Extracellular apparent K^+^ affinity of the ATPases: current-voltage relationships of wild-type (**i**) and mutant pumps (**ii**) in various [K^+^]_o_ conditions normalized to the outward current at +20 mV in 15 mM K^+^ (p.S779N *n = *5–6; wild-type *n = *3–5). (**E**) Concentration-response curves obtained from the data in **D** at three representative voltages. Dotted lines represent fits to a modified Hill equation (wild-type EC_50_ at −100, −60, 0 mV: 3.5 ± 0.3, 2.2 ± 0.2, 1.3 ± 0.1 mM, *n = *5; *P < *0.0001 repeated measures ANOVA; p.S779N EC_50_ at −100, −60, 0 mV: 6.9 ± 0.4, 6.9 ± 0.2, 7.5 ± 0.7 mM, *n = *5; *P = *0.7). Curves obtained from separate experiments were normalized to the top and bottom values of the fit and averaged across oocytes. Hill coefficients were voltage independent and did not change significantly between pump variants: wild-type 1.54 ± 0.06, S779N 1.56 ± 0.06 (*P = *0.3, two-way ANOVA across the voltages). (**F**) Overall voltage dependence of apparent [K^+^] affinity (wild-type *n = *3–5; p.S779N *n = *–5, *P < *0.001 two-way ANOVA).

The forward pumping activity of the Na^+^/K^+^-ATPase was initially studied in 15 mM [K^+^]_o_ that saturates the wild-type turnover rate (DiFranco *et al.*, 2015). The outward pump current was reduced in p.S779N compared to the wild-type ATPase (Fig. 3A and B, *P < *0.0001). Normalization of steady state currents to total Na^+^ charge movement confirmed a lower turnover rate at voltages positive to −40 mV (Fig. 3C, *P < *0.001). To test if the reduced pump currents result from altered K^+^ affinity, we studied pump currents in various [K^+^]_o_. Currents did not saturate for the p.S779N pump, even with 30 mM [K^+^]_o_, while the wild-type pump currents activated and saturated at lower [K^+^]_o_ (Fig. 3D). The wild-type pump showed a depolarization-induced increase in K^+^ affinity while the EC_50_ of p.S779N was largely voltage-independent (Fig. 3E) and was higher across all voltages (Fig. 3F, *P < *0.001).

Furthermore, in 2–5 mM [K^+^]_o_ the net current in the mutant pump is inward at hyperpolarized voltages (Fig. 3D), suggesting that the aberrant leak current persists at physiological [K^+^]_o_ and may interfere with the accurate estimation of forward pump currents.

#### Ionic nature of the leak current

**Figure 4:**
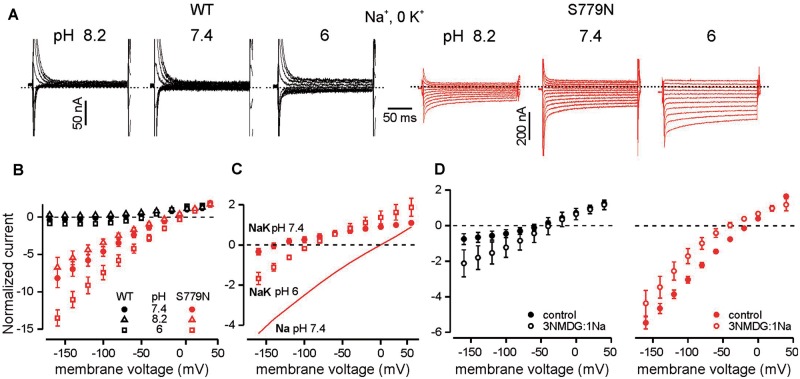
**Ionic contributions to the p.S779N ATPase leak current.** (**A**) Representative current traces in 0 [K^+^]_o_ for wild-type (black) and p.S779N (red) illustrating changes in leak currents with varying [H^+^]_o_. (**B**) Steady state current-voltage relationships in different pH conditions normalized to current at +20 mV in pH 7.4 (*n = *4 for both). Linear fits to raw leak currents (not shown) in p.S779N indicate an increase in slope conductance with acidification from 0.54 ± 0.1 to 0.85 ± 0.2 nA/mV (*P = *0.03, paired *t*-test) and a shift in E_rev_ from −12.7 ± 2.5 to −2.2 ± 2.6 mV (*P = *0.01). For pH 8.2 the slope was 0.43 ± 0.1 (*P = *0.15) and E_rev_ = −17.8 ± 2.2 mV (*P = *0.06). (**C**) Increase in inward leak currents in p.S779N pump with acidification in the presence of 15 mM [K^+^]_o_; comparison with linear leak in Na^+^-only (pH 7.4) conditions (*n = *4). Currents have been normalized to amplitude at +20 in pH 7.4. (**D**) Average steady state currents for wild-type (*left*) or p. S779N (*right*) pumps in control conditions (115 mM Na^+^, 0 K^+^) and when 86 mM Na^+^ is substituted with NMDG^+^. Currents have been normalized to control value at +20 mV (wild-type *n = *6, *P = *0.008; p.S779N *n = *7, *P = *0.001 repeated measures ANOVA). For the mutant pump, E_rev_ shifted from −16.2 ± 1.5 mV in Na^+^ to −48.8 ± 10 mV in Na/NMDG^+^ (*n = *7, *P = *0.016, Wilcoxon paired *t*-test).

The ionic species carrying the inward leak of the p.S779N pump were investigated in absence of forward pumping currents in 0 [K^+^]_o_. Lowering external pH to 6 augmented the leak current and right-shifted its reversal (Fig. 4A and B; slope: *P = *0.03, E_rev_: *P = *0.01) while increasing pH to 8.2 had the opposite effect but without statistical significance (slope: *P = *0.15, E_rev_*P = *0.06). Changing pH was of little consequence for the wild-type pump (Fig. 4A and B). These results indicate that protons contribute to the leak in the mutant Na^+^/K^+^-ATPase. Furthermore, reduction of pH to 6 in the presence of 15 mM [K^+^]_o_ altered p.S779N pump currents in a manner consistent with an increased linear leak component (Fig. 4C), suggesting that the leak current is present even in high [K^+^]_o_. This also suggests that the current measured in presence of [K^+^]_o_ is in fact the sum of forward pumping current and the leak current and consequently that the estimation of [K^+^]_o_ dependence of the outward current is interfered by the presence of the leak current.

When 75% of the extracellular Na^+^ was replaced with NMDG^+^, the inward leak current of the mutant Na^+^/K^+^-ATPase was reduced (Fig. 4D, *P = *0.001) along with a negative shift in reversal potential (*P = *0.016). In contrast, the substitution enhanced steady state currents in the wild-type pump (Fig. 4D, *P = *0.008). When extracellular Na^+^ was elevated from 115 mM to 145 mM the slope of the linear leak current increased and reversal potential shifted towards positive voltages for p.S779N pumps (Supplementary Fig. 1, *n = *4, *P < *0.05 for both slope conductance and reversal potential). These data point to an additional contribution of Na^+^ ions to the leak current of p.S779N pump.

Finally, as there is controversy in the literature whether mutation of the serine reduces intracellular sodium affinity or not (Argüello and Lingrel, 1995; Blostein *et al.*, 1997; Pedersen *et al.*, 1998), we studied the leak currents of p.S779N without prior loading of the oocytes. Loading has been shown to increase the intracellular sodium concentration and thereby stimulate forward pump current of Na^+^/K^+^-ATPases, depending on the sodium affinity of the pump expressed (Horisberger and Kharoubi-Hess, 2002). Consistently, we measured small or no potassium-induced forward pumping for p.S779N and only small currents for wild-type pumps in unloaded oocytes. The amplitudes of the charge carried were also markedly reduced for both p.S779N and wild-type pumps compared to loaded oocytes (Supplementary Fig. 1B), and no leak currents were detected for p.S779N in unloaded oocytes (Supplementary Fig. 1C).

## Discussion

We have found the p.S779N variant of the Na^+^/K^+^-ATPase pump isoform 2 in a patient presenting with hypoPP and CNS symptoms. No mutations were detected in the known hypoPP genes. The mutation is absent in exome databases (gnomAD) and in the asymptomatic parents, suggesting it had arisen *de novo.* This genetic evidence supports association of the variant with the clinical presentation and the symptoms of the patient are consistent with the expression of Na^+^/K^+^-ATPase α2 pump in the skeletal muscle and the glia. Functional analysis revealed a leak current in the mutant pump, analogous to gating pore currents carried by mutant voltage gated sodium and calcium channels. These currents underlie hypoPP (Cannon, 2015). The depolarizing inward leak currents in the mutant pump were measured only at hypokalaemic conditions, consistent with the low serum potassium measured in the patient during symptoms. Thus, the functional evidence strongly supports the association of the variant with periodic paralysis in the patient.

Similar to the gating pore leak currents through voltage gated ion channels associated with hypoPP the inward leak current can be carried by Na^+^ ions and protons. In muscle, proton influx leads to increased intracellular Na^+^ accumulation through action of the Na^+^/H^+^-exchanger (Jurkat-Rott *et al.,* 2009), suggesting that the leak may cause muscle depolarization by promoting (directly and indirectly) influx of different cation species to muscle fibres.

Wild-type Na^+^/K^+^-ATPase α2 pump current density in muscle is ∼1 µA/cm^2^ at −90 mV in 10 mM [K^+^]_o_ (DiFranco *et al.*, 2015). Considering that the turnover rate of the mutant pump is reduced compared to wild-type and by comparing the relative amplitudes (at −80 and −100 mV) of p.S779N inward leak in low [K^+^]_o_ and outward current in high [K^+^]_o_, we can estimate that the inward current amplitude of the mutant pump in 2 mM [K^+^]_o_ is between one-third and one-half of the outward wild-type currents at −90 mV measured by (DiFranco *et al.*, 2015). Assuming equivalent allelic expression of p.S779N and wild-type pumps in heterozygous condition in the muscle, this would result in a leak current density of ∼0.17–0.25 µA/cm^2^. This value is close to the measured gating pore current density in a mouse model of hypoPP (0.2 µA/cm^2^, Wu *et al.*, 2011). Despite its small amplitude, this current is sufficient to cause muscle depolarization particularly in the presence of hypokalaemia, which substantially reduces outward K_ir_ current in skeletal muscle (Cannon, 2015).

However, it is important to stress that the estimated current amplitude is based on heterologous expression in the absence of any additional pump subunits expressed in skeletal muscle, such as FYXD1, in lower extracellular Na^+^ concentration (Supplementary Fig. 1A) than in muscle and on recordings at room temperature, although temperature is not expected to change the proportion of leak and pump current (Meier *et al.*, 2010). FYXD1 has been shown to reduce the affinity of the α2 pump for external potassium (Han *et al.*, 2009; Stanley *et al.*, 2015) and would be anticipated to increase the proportion of the leak current. These factors suggest that the precise relation of the wild-type forward pump current and the mutant inward leak current in muscle may differ from that in our recordings. Similar expression levels of wild-type and mutant pumps measured as total transient Na^+^-dependent currents in *Xenopus* oocytes suggests that the membrane stability of mutant pumps is not reduced in this system. Altogether these data suggest that the amplitude of the p.S779N leak current is in the same range as the hypoPP-associated gating pore currents and can induce muscle depolarization, strongly supporting the association of the mutation with hypoPP.

Many of the ∼80 mutations in *ATP1A2* associated with CNS phenotypes (Bøttger *et al.*, 2012; Pelzer *et al.*, 2017) reduce the turnover rate of the α2 Na^+^/K^+^-pump without causing skeletal muscle presentations. This suggests that the leak current, rather than the reduced turnover rate, is the main pathomechanism of hypoPP in our patient. Two C-terminal mutant α2 Na^+^/K^+^-ATPases with only reported CNS presentations do also conduct leak currents (Poulsen *et al.*, 2010). However, these leak currents in the absence of K^+^ present at voltages negative to −100 mV, which means they are not active at resting membrane voltage. These data further highlight our proposition that it is the loss-of-function features of these mutants that underlie the neurological phenotype in these mutants and in our p.S779N patient.

Similar leak currents have been detected in α1 Na^+^/K^+^-ATPase mutant pumps found in patients with hyperaldosteronism (Meyer *et al.*, 2017). In this case the leak current amplitude at physiological voltages was not sufficient to result in an inward net current suggesting that the leak current does not contribute to the depolarization in these cells. Rather, the authors suggest loss-of-function mechanism is common cause for hyperaldosteronism (Meyer *et al.*, 2017).

Although with a single case we cannot completely exclude chance association of the p.S779N variant with hypoPP in this patient, the outstanding difference between the functional features of p.S779N and the FHM2-associated mutations—i.e. inward cation leak at voltages near rest—strongly supports the notion that p.S779N anomalous steady state currents underlie membrane depolarization and muscle inexcitability leading to paralysis in patients with hypoPP. Thus, our data provide important evidence supporting a leak current as the major pathomechanism underlying hypoPP and indicate *ATP1A2* as a new hypoPP gene.

Small proton leak currents are an inherent property of the transport cycle of wild-type Na^+^/K^+^-ATPases, being more pronounced in 0 [Na^+^]_o_ and low pH, and effectively inhibited by forward pumping in saturating [K^+^]_o_ (Wang and Horisberger, 1995; Horisberger and Kharoubi-Hess, 2002; Vasilyev *et al.*, 2004; Poulsen *et al.*, 2010; Mitchell *et al.*, 2014; Vedovato and Gadsby, 2014; Hilbers *et al.*, 2016). We found an abnormally large leak current in p.S779N, both in low and high [K^+^]_o_. The leak can outbalance the pump current at physiological voltages when [K^+^]_o_ or pH are reduced. Normal leak currents are associated with the molecular conformations where extracellular Na^+^ is released, the so-called E2P conformation (Li *et al.*, 2006; Poulsen *et al.*, 2010; Vedovato and Gadsby, 2014; Stanley *et al.*, 2016; Isaksen *et al.*, 2017). The relative stabilization of this conformation coupled with a profoundly reduced K^+^ affinity in the mutant p.S779N pump likely contribute to the presence of an aberrant leak current.

Consistent with a key location of the S779 residue on the highly conserved K^+^ binding site of the enzyme (Morth *et al.*, 2007; Fig 1), the p.S779N mutation increases the K^+^ EC_50_ of the pump 2–6-fold. In agreement with our results, when sheep α 1 S775, analogous to α 2 S779, was mutated to either alanine, cysteine or tyrosine, the mutants had markedly reduced affinity for [K^+^]_o_ (Argüello and Lingrel, 1995; Blostein *et al.*, 1997; Pedersen *et al.*, 1998). In addition to inducing forward pumping, the effect of [K^+^]_o_ likely includes inhibition of the leak current, which may affect the estimation of K^+^ affinity of the forward pumping. Our data indicate that the leak current is present even at 15 mM [K^+^]_o_ (Fig. 4C). Although we cannot discern between the two effects of K^+^, if both arise from the same K^+^ binding step, then the EC_50_ gives an accurate measure of reduced K^+^ affinity of the pump. Inhibition of the leak current by elevated [K^+^]_o_ may explain the beneficial clinical effect of K^+^ supplements in the patient. In normokalaemic conditions the mutant pump does not carry a net inward current that would lead to depolarization of the cell and hypoPP. However, an inward leak of H^+^ during forward pumping may contribute to the clinical presentation even in normokalaemia, in particular upon extracellular acidosis. Reduced net outward current in mutant pump compared to wild-type pump activity in normokalaemia is qualitatively similar to loss of function mutation associated with FHM and is likely to contribute to the CNS presentation.

Moreover, the left-shifted voltage dependence of transient Na^+^-dependent currents indicate a reduced [Na^+^]_o_ affinity of p.S779N α2. Different mutations of α1 S775 have been shown to reduce [Na^+^]_i_ affinity 8–14-fold (Pedersen *et al.*, 1998), not affect it (Argüello and Lingrel, 1995) or reduce it slightly by 1.5-fold (Blostein *et al.*, 1997). Therefore, we tested p.S779N in oocytes without initially raising the [Na^+^]_i_, and detected no leak currents under these conditions. The low intracellular sodium level also restricted forward currents from both the mutant and wild-type pumps, suggesting that they become halted in the catalytic cycle at E1. Reduced [Na^+^]_i_ affinity of p.S779N may therefore limit it from entering the E2P state(s) that are generally suggested to be associated with conducting inward leak currents (Nyblom *et al.*, 2013; Stanley *et al.*, 2016). Consequently, it is feasible that the leak currents are only physiologically relevant at elevated intracellular sodium concentrations, for example [Na^+^]_i_ can increase 3-fold upon electrical stimulation (Fong *et al.*, 1986). In the patient, the effect would be a negative loop where high intracellular sodium levels would be even further increased by the mutant pump, counteracting the actions of the pump from the wild-type allele.

The CNS symptoms in the proband are similar to the epileptic and cognitive phenotypes observed in some FHM2 and alternating hemiplegia of childhood patients carrying loss-of-function mutations in α2. This suggests that the reduced Na^+^ and K^+^ turnover of the p.S779N pump contributes to CNS dysfunction. The α2 Na^+^/K^+^-ATPase specializes in fast K^+^ clearance from the diffusion-restricted spaces of the T-tubules (DiFranco *et al.*, 2015) and synapses (Larsen *et al.*, 2014), where K^+^ concentrations rise above 10 mM during periods of activity. Reduced astrocytic K^+^ and Na^+^ pumping may therefore lead to synaptic K^+^ accumulation and disruption of Na^+^-dependent secondary transport processes, such as glutamate clearance (Illarionova *et al.*, 2014) and Ca^2+^ signalling (Golovina *et al.*, 2003), thereby contributing to the CNS phenotype. The role of the p.S779N leak current in astrocyte function remains to be investigated.

### Clinical implications

Our data implicate *ATP1A2* as a new hypoPP gene and leak currents as a common pathomechanism of hypoPP. To our knowledge this is the first report of a mutation in a non-channel gene resulting in a leak current associated with hypoPP. Since at least 10% of hypoPP cases are genetically undefined, we propose that genetic screening, in combination with functional characterization, may link further cases with mutations in *ATP1A2* and potentially other genes encoding transmembrane proteins expressed at similar levels in skeletal muscle.

No cardiac presentations have been reported for the *ATP1A2* variants associated with CNS and skeletal muscle symptoms despite expression of α2 in the heart. In this tissue, however, this isoform accounts for <20% of the total Na^+^/K^+^ ATPase contents (as opposed to ∼85% in skeletal muscle) (Orlowski and Lingrel, 1988; Berry *et al.*, 2007; Radzyukevich *et al.*, 2013). Hence, the detrimental effects of the leak current and loss-of-function properties of p.S779N in the heart may be negligible. Lack of cardiac manifestations may also arise due to differential localization or regulation of the pump in cardiac muscle (Juhaszova and Blaustein, 1997), or to tissue specific compensatory mechanisms for the effects of *ATP1A2* mutations such as different ion channel expression pattern. Notwithstanding these facts, it is reasonable to suggest that patients carrying *ATP1A2* variants may be monitored for cardiac dysfunction, in addition to skeletal muscle dysfunction.

Acetazolamide is a common treatment for hypoPP (Matthews *et al.*, 2011). It causes metabolic acidosis predicted to increase the leak current through the p.S779N pump, consistent with acetazolamide worsening muscle symptoms in our patient. This implies that acetazolamide should be avoided in cases associated with *ATP1A2* mutations that result in H^+^-carried leak currents. Instead, our findings suggest that increasing [K^+^]_o_ with K^+^ supplements or K^+^-sparing diuretics will boost pump activity, promoting net outward currents through the p.S779N pump and compensating the inward leak. This may reduce the incidence of episodes of paralysis.

In summary, we present a child with hypoPP carrying a novel mutation in *ATP1A2.* CNS symptoms are also present, distinct from other hypoPP cases. Electrophysiological studies of the p.S779N mutant pump reveal an anomalous inward leak current and altered turnover rates, providing a mechanistic explanation for the periodic paralysis and CNS symptoms, respectively. Our results indicate that *ATP1A2* is a new hypoPP gene.

## Funding

This work was supported by an MRC Centre grant (MR/K000608/1), MRC project grant (MR/M006948/1), a Wellcome strategic award, and the UCLH NIHR Biomedical Research Centre. EM is supported by a Wellcome Clinical Research Career Development Fellowship.

## Competing interests

The authors report no competing interests.

## Supplementary Material

Supplementary Figure S1

## Abbreviation

hypoPP: = hypokalaemic periodic paralysis
