# Supplementary material for: A novel ATP1A2 mutation in a patient with hypokalaemic periodic paralysis and CNS symptoms
Source: Brain. 2018 Nov 12;141(12):3308–18. doi: 10.1093/brain/awy283 (PMC6262219; doi:10.1093/brain/awy283)
Supplement: Supplementary Figure S1 [file awy283_supplementary_figure_1.pdf]

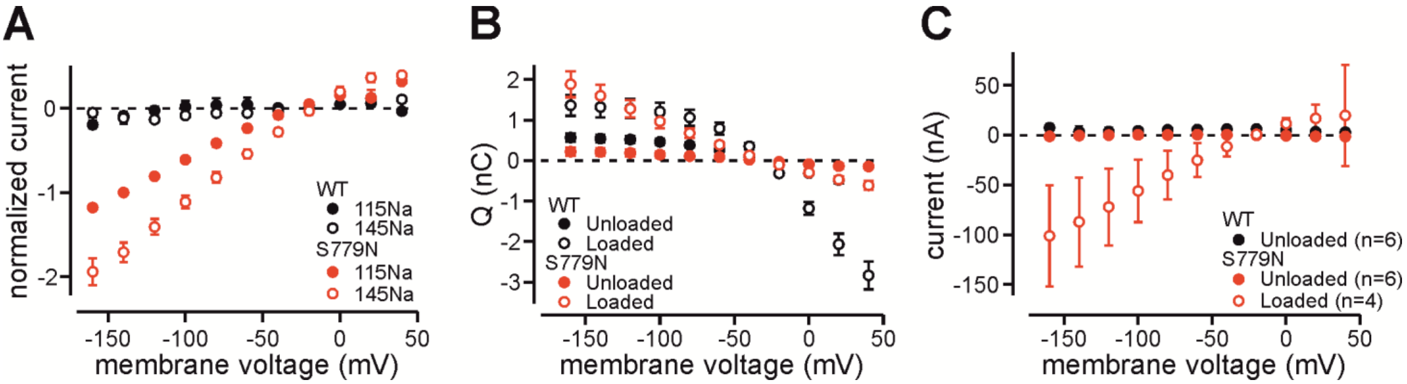

Supplementary Figure 1. A. Comparison of leak current amplitude in 115 mM and 145 mM extracellular Na^+. Data for p.S779N (n=4) are normalised to current amplitude in response to voltage step to -140 mV measured in 115 mM Na^+. Wild type data (n=4) is normalised by comparing the mean current amplitudes of wild type and p.S779N pumps at -140 mV and dividing the wild type currents with the fold difference. B. Charge-voltage relationships measured in loaded (open symbols) and unloaded (solid symbols) oocytes. The dataset for loaded oocytes is from experiments that were used to estimate the Q_{tot} in figure 3C. C. Current-voltage relationships of the steady state current in loaded (open symbols) and unloaded (solid symbols) oocytes. Data for only a subset of loaded oocytes that were studied at the same batches of oocytes as the unloaded oocytes are shown.
